# Microwave Plasma-Enhanced Parylene–Metal Multilayer Design from Metal Salts

**DOI:** 10.3390/nano12152540

**Published:** 2022-07-24

**Authors:** Mirco Weber, David Vorobev, Wolfgang Viöl

**Affiliations:** 1 Faculty of Enginering and Health, HAWK University of Applied Sciences and Arts, Von-Ossietzky-Straße 99/100, 37085 Göttingen, Germany; mirco.weber@hawk.de (M.W.); david.vorobev@hawk.de (D.V.); 2 Institute of Inorganic Chemistry, Georg August University of Göttingen, Tammannstraße 4, 37077 Göttingen, Germany; 3 Fraunhofer Institute for Surface Engineering and Thin Films, Application Center for Plasma and Photonic, Von-Ossietzky-Straße 100, 37085 Göttingen, Germany

**Keywords:** Parylene C, Parylene multilayer, barrier coatings, chemical vapour deposition

## Abstract

In this paper, a new approach for the synthesis of Parylene–metal multilayers was examined. The metal layers were derived from a metal salt solution in methanol and a post-drying plasma reduction treatment. This process was designed as a one-pot synthesis, which needs a very low amount of resources and energy compared with those using electron beam sputtering processes. The Parylene coatings were obtained after reduction plasma treatments with Parylene C. Therefore, a Parylene coating device with an included plasma microwave generator was used to ensure the character of a one-pot synthesis. This process provided ultra-thin metal salt layers in the range of 1–2 nm for layer thickness and 10–30 nm for larger metal salt agglomerates all over the metal salt layer. The Parylene layers were obtained with thicknesses between approx. 4.5 and 4.7 µm from ellipsometric measurements and 5.7–6.3 µm measured by white light reflectometry. Tensile strength analysis showed an orthogonal pulling stress resistance of around 4500 N. A surface roughness of 4–8 nm for the metal layers, as well as 20–29 nm for the Parylene outer layer, were measured. The wettability for non-polar liquids with a contact angle of 30° was better than for polar liquids, such as water, achieving 87° on the Parylene C surfaces.

## 1. Introduction

The sustainable sealing of component parts or electronic components that are heavily stressed by external influences plays a major role in many areas, such as the electrical industry, sensor technology, space travel, electro-optical assemblies, and many more. This sealing to protect against external influences is generally called barrier coating. Such barrier coatings can be achieved in different ways. A widespread method for barrier coating applications is to seal with polymers from the liquid or gaseous phase or lacquers. The advantages of this method are the low process costs and the often-simple handling and applicability. However, this method also has the disadvantage that a layer thickness in the range of 10–100 µm is required for effective barrier sealings [1,2].

As a result, precisely shaped components lose their fitting accuracy, and contour sections that may be important for installation could be blurred. Further disadvantages are, on the one hand, that optical signal transmission is affected by the high coating thickness, and, on the other hand, that the high, potentially toxic exposure to solvent vapours can have health consequences. Therefore, an alternative is low-pressure chemical vapour deposition (CVD) with Parylene [3]. This is a semicrystalline coating material that partially alternates between aromatic and aliphatic parts, and forms barrier properties in the single-digit micrometre range [4]. Dependent on the pressure at the CVD process, Parylene coatings vary in their crystallinity and stiffness [5]. Crucial application fields are the protection of biomedical implants in the aspect of corrosion resistance [6] and usage as an electric insulation due to its dielectric properties [7]. A big disadvantage, however, is that barrier sealing with Parylene does not allow any signal transmission except for optical signals outside the layer absorption range [8,9,10,11,12,13]. Furthermore, light transmission in certain wavelength ranges can also become problematic if the substrate to be protected is sensitive to certain wavelengths. To compensate these problems, research for the modification possibilities of Parylene layers by creating a multilayer coating system is of great interest. For example, the combination of Parylene layers with other materials in the field of biomedical implants is a great advantage because small transparent, flexible and stretchable electronic devices can be fabricated. Von Metzen et al. [14] demonstrated a multilayer system on this topic, which consisted of two 10 µm thick layers of Parylene C with enclosed platinum conductor structures with a thickness of 300 nm. One possible application is the implementation in the process structure of microelectromechanical systems (MEMS) in order to ultimately gain access to new implant technologies. 

Hogg et al. [15] found that a key advantage of Parylene-based implants was the protection of the implant from body fluids and the protection of the body from toxic components of the implant using a comparatively thin layer thickness. Furthermore, a multilayer system was presented in that study, which provided an increased barrier effect. It was created through a combinable process within a process chamber by thermal and plasma-assisted chemical vapour deposition, incorporating SiO_X_ components into the Parylene layers. Kuo et al. [16] also worked on the incorporation of alternating SiO_X_ and SiN_X_ layers in the order of 50 nm per layer to improve the barrier protection of Parylene layers for implant materials. In the long term, these biomedical implants are intended to provide support in the cerebral area; for example, to correct faulty bioelectrical signal transmissions by means of targeted electrical impulses. Due to the complex geometric conditions within the human body, and also brain, the use of inflexible implants with little movement is extremely difficult. Therefore, *Kwon* et al. [17] researched a Parylene multilayer implant solution to overcome these difficulties. The use of such flexible technologies in the brain, for example, can alleviate neural diseases, such as Parkinson’s, or even certain physical impairments. Parylene–metal multilayer constructs can be used in retinal implants to act as wireless current transfer agents [18] to facilitate drug delivery, or to stimulate nerves to compensate for specific conditions in this area as well. However, useful fields of application of Parylene-based multilayer systems are not limited to the medical sector; further usages in the electronic and sensory fields are also possible. For example, Yang et al. [19] used Parylene–metal multilayers as a membrane component of ultrasonic transducers to implement a thin-film barrier isolation option in miniature devices. In general, Parylene in combination with metal layers are used as flexible [20,21,22,23,24], stretchable [21] and light transmissive [21,25,26] matrices, and as protective [25,26,27], isolating [28,29,30] and dielectric material [24,31,32,33] for OLED design [26,29], organic solar cells [25], microelectronic devices [20,22,24,28,31,33], prostheses [22], implants [34] and coplanar waveguides [32]. Those systems are also reported to improve the fracture resistance in dental ceramics and composite materials [27]. 

It is known that transition metals and their salts can inhibit the deposition of polymeric Parylene films on substrate surfaces by one-electron-reduction between radical polymer chains and transition metals [35]. However, this also provides new application possibilities. Vaeth et al. [36] used this inhibition effect to implement a selective chain growing process within the Parylene deposition by the use of different transition metal compounds. It was shown that the inhibition potential directly correlated with the formation of nucleation sites. Another usage of Parylene–metal salt interaction was the creation of differently structured polymer surfaces in combination with organic thiols [37]. 

In many multilayer systems, elementary metal layers or structures have been produced in the nanometre range by sputtering processes or electron beam deposition. Such thin layers give access to new applications in the field of electrical devices on smaller scales and in geometries that are difficult to access. Furthermore, they save considerable amounts of material and also produce new properties. For example, layers of gold nanoparticles can also exhibit plasmon resonant effects, whereby analytical and sensory tasks can be performed in addition to the usual electrical signal transmission functions. This allows the development of metal thin-film multilayer generation suitable for future applications in the microelectronics field, to meet further challenges. However, deposition via sputtering systems involves increased operational, energetic, equipment-related, and thus financial costs. Therefore, this study explores the possibility of accessing metallic thin films in the nanometre range at a lower cost by modifying and modelling the plasma treatment option of a conventional Parylene coating system in the sense of a one-pot synthesis from metal salt layers, in order to demonstrate an overall more cost-effective alternative.

## 2. Materials and Methods

### 2.1. Sample Preparation

The plasma and coating experiments were carried out using the LAB Coater 300 LV 35 RR (*Plasma Parylene Systems GmbH,* Rosenheim, Germany) with a volume of approx. 40 L inside the coating chamber, which also corresponded to the volume of the gas discharge of the low-pressure plasma used. This coating system operated at pressures of 4–10 Pa and consisted of a coating unit with integrated evaporation and pyrolysis device, as well as a coating chamber with a microwave generator. The microwave generator ignited a low-pressure plasma inside the coating chamber. A cold trap with a connected rotary vane vacuum pump generated the vacuum required for coating and plasma treatment. The process gas for plasma treatment was Varigon gas (*Linde AG*) consisting of 95% argon and 5% hydrogen. 

**Figure 1:**
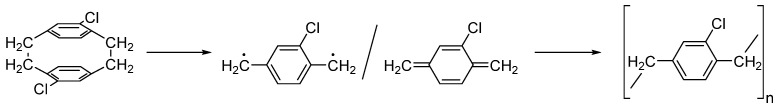
Own representation of the polymerisation reaction to obtain Parylene C.

The plasma treatments were carried out at a microwave frequency of f = 2.45 GHz and a microwave power of P = 850 W. This corresponds to a power density of 21.25 W/L. These parameters were found to be the most suitable to ignite hydrogen for reduction purposes. Using internally installed mass flow controllers, the gas flow rate was set to 1000 sccm. The coatings were applied with Parylene C. The powdered precursor was introduced into the evaporator unit, evaporated at a temperature of 110–120 °C and pyrolysed at 720 °C. The coating deposition onto the substrates was conducted at about 40–45 °C. A simplified reaction scheme of the Parylene C polymerization as an own representation based on the research of Fortin et al. [38] is shown in Figure 1. All coatings generated for this study took place in a pressure range between 9 and 11 Pa and were carried out with a precursor quantity of 10 g to obtain a layer thickness of around 5 µm, which has found to the most suitable for analytical purposes. 

To generate the multilayer coating systems, different sample types were created, each representing specific production steps, thus enabling a more in-depth analysis, a better understanding of the different components and their interaction with each other in the coating system. The used sample types are defined for this purpose in Table 1.

In this respect, iron(III) chloride (FeCl_3_), copper(II) chloride (CuCl_2_) and iron(II) sulphate (FeSO_4_) were selected for this study. This metal compounds have different stable contiguous oxidation states which can be altered by plasma reduction. FeSO_4_ has the advantage that this compound can be treated both reductively and oxidatively. All three compounds were treated in a Varigon low-pressure plasma for 30 min under the given parameters. The application followed a specific reproducible scheme. First, metal salt concentrations in a suitable solvent were determined. The solvent in this study was methanol, as its lower boiling point makes it easier to remove than water. The concentrations used in total were 0.29 mol/L, 0.15 mol/L, 0.07 mol/L, 0.04 mol/L, 0.02 mol/L and 0.01 mol/L. For the ellipsometric analysis, concentrations from 0.04 mol/L to 0.29 mol/L were used to verify a possible dependence on the formed metal salt layer thickness by the applied concentration. The solutions with concentrations of 0.02 mol/L and 0.01 mol/L were defined as solution 1 and solution 2. These two concentrations were chosen to maintain the transparent character of the glass slide substrates, whereas coating with solutions of higher concentrations led to relevant turbidity due to the stronger formation of metal salt agglomerates. Thus, samples with a coating of solution 1 and solution 2 served as representative samples for the multilayer coating system, and the solutions with higher concentrations for better handling in specific analytical procedures. The intention was to create metal salt layers as thin as possible so that a plasma treatment directly affected most of the layer materials. On the other hand, for most applications of Parylene–metal multilayers, the metal layer thickness was usually in the nanometre range. It was considered best to obtain a thickness in the low nanometre scale to preserve a wide application field and to ensure a plasma impact as effective as possible. However, the drop-on procedure also required process uniformity to ensure reproducibility in the production and statement of the analytical results. 

The procedure was standardised to a specific drip volume and application method by applying a defined drop volume of the metal salt solutions, averaging 19 µL per drop. Five drops, applied at even intervals, were used for application to the glass slides. To ensure better distribution, a second glass slide was placed flush after dripping so that the solution could spread over the entire area between the slides. For drying, the superimposed glass slides were separated, placed in the low-pressure coating chamber, and dried at 5–10 Pa for 30 min. Subsequently, further steps such as plasma treatments and/or coatings could be carried out with these samples.

### 2.2. Surface Analysis and Evaluation

Both a white light reflectometer consisting of a halogen spectral lamp with a *TranSpec MC-UVNIR-H* spectrometer (range: 190–1020 nm) and an *EP4 ellipsometer* (from *Accurion GmbH*) were used to analyse the layer thicknesses. The optical inspection of the coatings was carried out with a digital microscope (*VHX 6000* from *Keyence Corp.*). The surface wetting properties were determined with a Mobile Surface Analyzer handheld device (*MSA*) from *Krüss GmbH* using the test liquids water and diiodomethane (1 µL per drop each). Atomic force microscopy (AFM) measurements and corresponding calculations were made with an *Easyscan 2* measurement module (*Nanosurf AG*). The area examined via AFM had a dimension of 50 × 50 µm^2^ and this area was scanned with a rate of 512 lines with a measurement time of 1.5 s for each line. Evaluations were carried out via the device control software of the *Nanosurf Easyscan 2*, whereby both the arithmetic mean and the mean over the least squares were taken into account in the roughness determination. 

## 3. Results and Discussion

### 3.1. Metallic Layer Modification

Attenuated total reflection infrared analysis (ATR-IR) of copper(II) chloride and iron(II) sulphate treated with Varigon plasma did not yield any results because the ratio of metal salt layer thickness and the thickness of the microscope cover glass was too large, so that only the glass was detected. For this reason, ATR-IR analysis with a thicker metal salt layer provided signals from this very layer, but no changes in the spectra of the samples could be detected over the plasma treatment times. This circumstance can be explained by the fact that the plasma effect only took place in the upper atomic layers of the metal salt [39,40]. Therefore, in the case of an increase in the metal salt layer thickness, a significant part of the layer material was not affected by the plasma treatment. Due to the comparatively high information depth of the ATR-IR analysis, the majority of the untreated layer material was detected, which meant that the final spectra did not show any changes. This meant that the ATR-IR analysis could be ruled out as a suitable means of identifying the plasma effect on the metal salt layer. 

### 3.2. Multilayer System Characterisation

**Figure 2:**
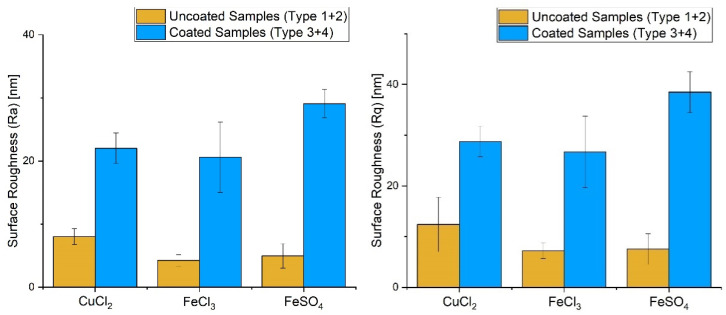
Comparison of surface roughness values between the different salt types.

In order to check the influence of the metal salt on sample types 1–4, several measurements via atomic force microscopy were made. The detailed results are given in the bar charts in Figure 2. It was found that the roughness of the glass slides was nearly identical for sample types 1 and 2, so the plasma treatment showed no effect. For this reason, the results were calculated and plotted together in Figure 2 and labelled “Uncoated Samples”. The roughness of the Parylene surface was also unchanged for the samples which were treated with plasma before Parylene coating compared with the untreated samples. This observation leads to the conclusion that the upper layers of the Parylene material were unaffected by previous Varigon plasma treatment. Due to that, the roughness measurement results were also combined in calculation and plotting with the label “Coated Samples”. Samples of types 1 and 2 were in the range of 4–8 nm for the arithmetic mean (*Ra*) and 7–12 nm for the least squares method (*Rq*). After Parylene treatment, the roughness was much higher. Thus, both with and without plasma treatment, sample types 3 and 4 showed an increase in roughness to *Ra* = 20–29 nm and *Rq* = 26–38 nm. It is noticeable here that for sample types 1 and 2, coating with iron salts FeSO_4_ and FeCl_3_ showed very similar roughness values. The samples from types 1 and 2 for CuCl_2_ had twice as much roughness as the iron samples. After Parylene coating, the roughness behaviour changed. For sample types 3 and 4, FeCl_3_ and CuCl_2_, i.e., the two chloride salts in the highest oxidation states, were approx. 20–22 nm for *Ra* and approx. 27–28 nm for *Rq*. Significantly higher roughness values were determined for FeSO_4_with approx. *Ra* and *Rq* = 29 and 38 nm, respectively.

**Figure 3:**
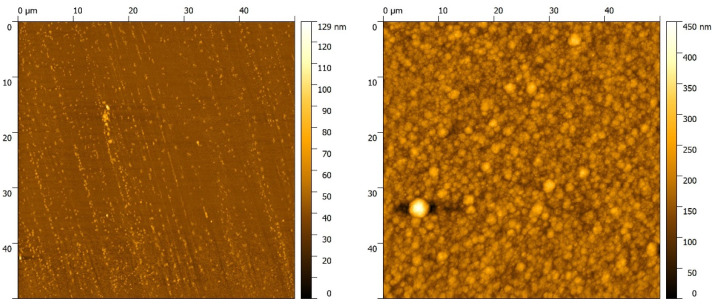

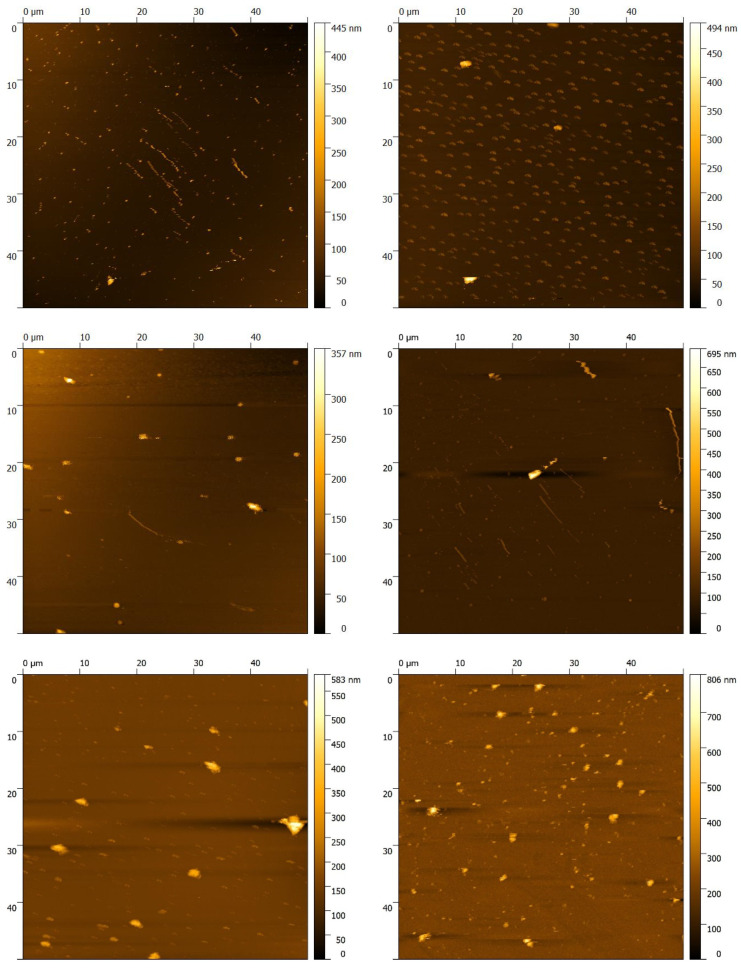
AFM images of a reference glass slide (**top left**), a Parylene-above-FeCl_3_ coated sample (**top right**), as well as the surface images of glass coated with CuCl_2_- (**2nd row**), FeCl_3_- (**3rd row**) and FeSO_4_- (**bottom**) layers before (**left**) and after plasma treatment (**right**).

In Figure 3, AFM images of the metal salt layers from sample types 1 and 2, the surface of the glass slide as a reference picture, and a coated example of an FeCl_3_ type 3 sample, are shown. It can be seen that the metal salt surfaces are populated with larger objects in micrometre size, as well as smaller ones in the sub-micrometre range. These larger formations were identified as crystalline structures with the help of microscopical examinations, such as those shown later in this study. A comparison between the AFM images of the type 1 and type 2 samples revealed that there were no clues of plasma etching detected. This observation confirms the conclusion drawn at the roughness measurements, that there were no significant plasma etching effects within the experimental setup of this study. 

For quality control of the coating deposition, the respective batch layer thickness was determined by means of white light reflectometry with the help of a reflective silicon reference surface. It was enclosed with the respective coating batch. The literature refractive index was used, since the layer thickness can only be calculated on the basis of the Fresnel equations with a known refractive index. During these measurements, it was observed that a layer thickness in the range of 4.579–6.304 µm was formed at a weight of around 10 g of the precursor for the coating with Parylene C. Thus, it can be concluded that per gram of Parylene C precursor weighed in, approx. 0.5–0.6 µm Parylene C layer thickness resulted. Another possibility of determining the layer thickness is the method of ellipsometry. With this method, even very thin layers can be measured without knowing their refractive index. These measurements (exemplary for the type 4 samples with CuCl_2_) confirmed, on the one hand, that the measurements by means of white light interferometry provided realistic results, and determined, on the other hand, how strongly the thickness of the metal salt layer formed with the same drop-on volume. By predetermining the Parylene layer thickness via white light reflectometry, the ellipsometric measurements over the visual to infrared spectral range (400–800 nm) could be carried out more time-efficiently, as a measurement in the UV range was no longer necessary.

The ellipsometric measurements showed a varying layer thickness between 1 and 30 nm. These values were derived from a specific sample spot. There were also larger crystalline structures, which could have a size in the micrometer range, as can be seen in Figure 3. In awareness of these crystals, the ellisometric measurement spots were chosen away from those. This procedure ensured that the ellipsometric modelling was in a realistic thickness regime. For most of the measurement spots, a metal salt layer of 1–2 nm was observed, but sometimes also some in the range of 10–30 nm. Due to the fact that every measurement showed a metal salt layer, these results were extrapolated to a usual layer thickness of 1–2 nm. Sometimes, aside from the observed crystal structures, there were agglomerations of metal salt material in the range of 10–30 nm. To compare the yield of coating thickness to the precursor weight, Table 2 and Table 3 show the results of the ellipsometric and white light reflectometry measurements.

**Figure 4:**
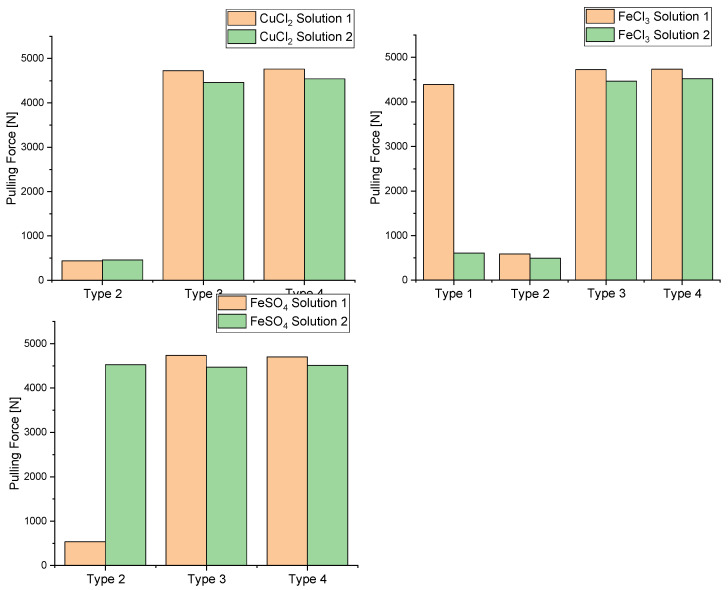
Comparison of the tensile tests between the different solutions and types with the salts CuCl_2_, FeCl_3_ and FeSO_4_.

In order to determine the strength of the laminated bond, the vertical pull-off force of aluminium test dollies, which were bonded to the type 1–4 specimens with an epoxy-based two-component adhesive, was determined. For this purpose, the *Positest Automatic Adhesion Tester* from the manufacturer *DeFelsko* was used, which consisted of a cylindrical pull-off tester for removing the dollies and a control unit for starting the measurement and displaying the stress–strain diagrams and the breaking force. The results of these measurements are shown in Figure 4.

During the peel tests, the metal salt and Parylene layers mostly broke, or, better, detached (adhesion breakage). However, complete destruction of the glass slides occurred in the samples of types 1 and 2. In a few samples without Parylene coating, the glass slides remained intact and an adhesive fracture (cohesion fracture) could be observed. This circumstance made it possible to determine the cohesive strength of the two-component adhesive at nearly 4500 N. For the other samples without Parylene coating, the destruction of the glass slides occurred at around 500 N. For the Parylene C coated samples of types 3 and 4, the adhesion failure also occurred at about 4500 N. Thus, the adhesive cohesion and the coating adhesion were in the same order of magnitude. Approximately the same values were obtained for both the samples with and without plasma treatment. This allows the conclusion that the treatment with a Varigon microwave plasma did not exert a significant influence on the layer adhesion of the individual sub-layers. From a chemical point of view, this observation also makes sense. Varigon gas consists of a gas mixture of hydrogen and argon. For plasma-enhanced adhesion improvement, an effective way is to create chemical bonding groups between the substrate material and the layer. These bonds are formed by linking two mostly polar functional groups. To generate such groups, the plasma discharge process gas should accordingly provide the components of these groups. For the generation of OH-groups, the process gas should contain certain proportions of oxygen and hydrogen. This can be achieved, for example, by using ambient air, as this contains both molecular oxygen and water due to the presence of humidity. However, neither component is present in the Varigon gas for the formation of such functional groups for the chemical bonding of the layer material to the substrate surface. This also explains the fact that there was no improvement in adhesion after plasma treatment. An adhesion improvement could be possible with the application of an oxygen-containing plasma, but this would also reoxidise the reduced metal salt surface of the interlayer. For this reason, a plasma-enhanced adhesion improvement was not an acceptable method for this study.

**Figure 5:**
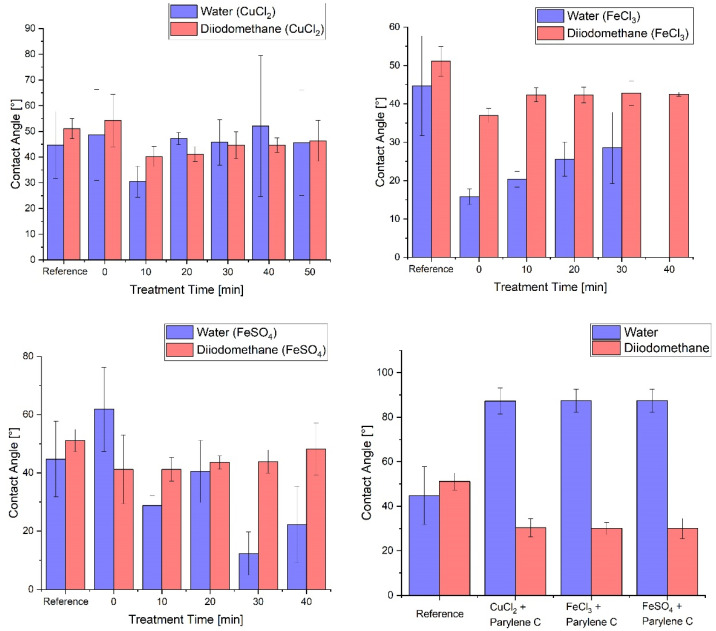
Results of the contact angle measurements with water for polar (blue) and diiodomethane for non-polar (red) liquids, with the contact angles of a glass slide as reference.

Furthermore, the wetting properties were investigated with the help of contact angle measurements. For this purpose, two liquids were used to analyse the wetting behaviour of polar and non-polar fluids. Deionised water was used as a representative of the polar fluid family and diiodomethane as a representative of non-polar fluids. When examining sample types 1 and 2, i.e., the samples with metal salt coating without a Parylene layer, a Varigon plasma with five different treatment times, namely, 10, 20, 30, 40 and 50 min, was used. The obtained results are displayed in Figure 5. However, there were no discernible deviations in the contact angles among the plasma treatments and in comparison, to the reference samples. This was due to the fact that the metal salt compounds were dissolved by the analysis liquids, which was observed after the contact angle measurements. On one hand, this changes the properties of the surface tension of the liquid droplets, and, on the other hand, the metal salt layer is significantly damaged by this dissolution process to such an extent that no polarity properties of this layer could be obtained. Particularly, the last two treatment times of 40 and 50 min for iron(III) chloride generated a water contact angle of 0°, because the droplet immediately spread out on the sample surface after the contact. This behaviour made it difficult to observe a plasma treatment time-dependent wetting capacity. As explained earlier, the contact angle measurement had a dissolving effect on the metal salt layer, due to the contact angles of 0°, which can be explained by the assumption that higher Varigon plasma treatment times lead to higher drying effects within the metal salt material. This could lead to a higher water absorption during the water droplet tests. The contact angle measurements of the samples coated with Parylene C gave a very solid picture of the polarity properties of the Parylene layers, which reflect a congruent wetting behaviour across all coated samples regardless of the metal salt type, as the Parylene layer was thick enough to exclude any influence of the associated metal compound. 

**Figure 6:**
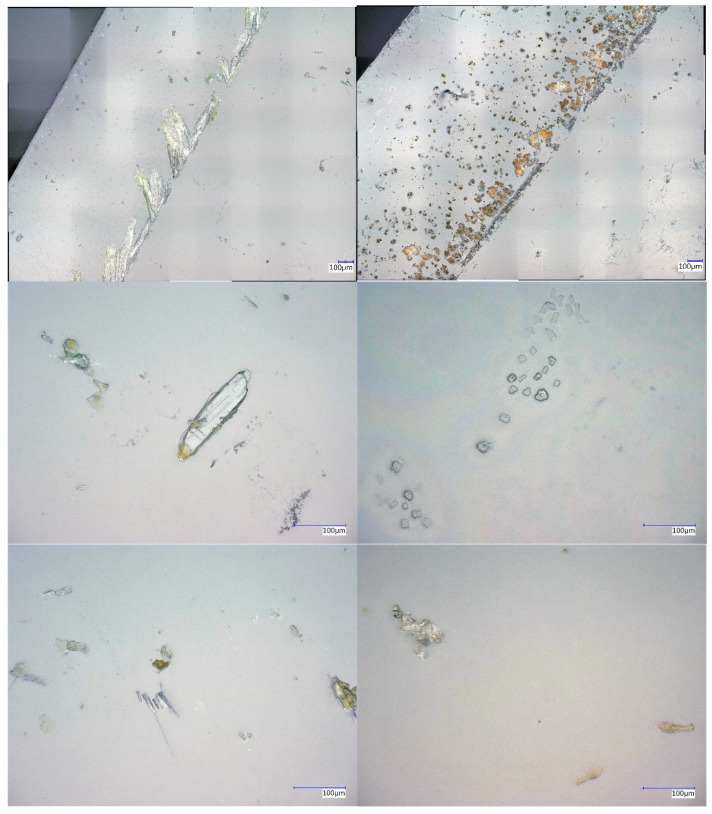
Microscopic images of metal salt layer from the edge (**top**) to the middle (**bottom**) of coated glass slides with CuCl_2_ (**left**) and FeSO_4_ (**right**).

In Figure 6, a more detailed overview of the layer distributions and characteristics on the surfaces of the glass slide substrates is shown by microscopic imagery. Random magnification images were taken at defined points on the samples using digital light microscopy. Here, the samples of types 1 and 2 provide insight into the layer expression of the metal salt layers. The comparison between these two sample types also shows the colour changes of the oxidised metal species after the application of a reductive Varigon plasma. When observing the images from the edge to the centre of the slide substrates, it is noticeable, in all samples, that larger crystalline agglomerates formed at the edge, while smaller crystalline agglomerates and powder-like equivalents appeared towards the centre. Colour changes in these agglomerates also provided information about redox-based changes within the metal layer material. The density of larger agglomerates decreasing towards the centre is an indicator that the support method, in which a glass slide dripped with metal salt solution is flush covered by another and thus weighed down, forces more metal salt solution outwards to the edges of the slide, as can be seen in Figure 6.

In addition, type 1 and 2 samples were found to have formed specifically elongated crystalline structures. A possible explanation for this behaviour could be the presence of small scratches on the glass slide surface. These scratches provided the forming crystal nuclei with an anchorage point, at which the critical mass of crystal nuclei, necessary for the onset of crystallisation, could be reached and exceeded. Thus, these small scratches offered a very attractive opportunity for crystallisation. A disadvantage of this observation is that thicker material appears to have formed there, which was only superficially affected by reductive plasma treatment. In general, thicker agglomerates should be avoided, as they cannot be completely brought to the desired oxidation state, and only the surface is influenced.

**Figure 7:**
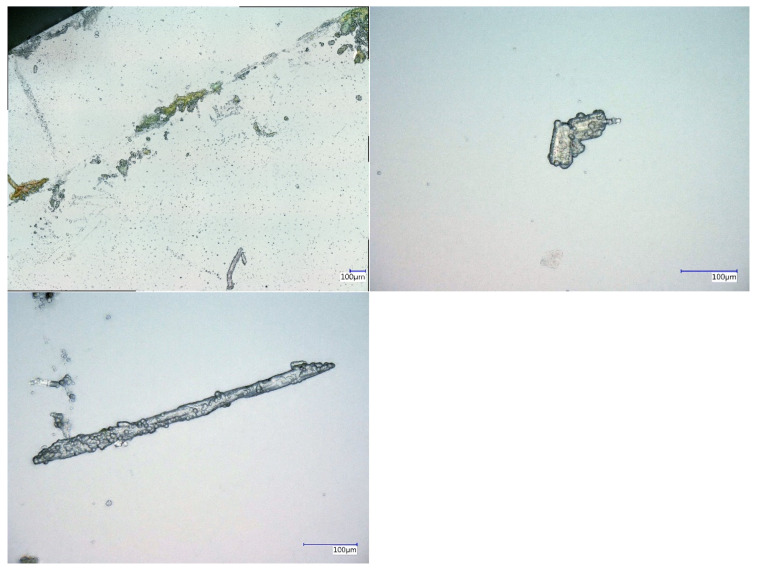
Microscopic images of CuCl_2_ and Parylene C coated samples with embedded gas bubbles between the Parylene matrix and the surface of crystalline agglomerates.

It could also be seen from the smaller crystalline deposits that the coating enclosed these structures, as shown in Figure 7. The conclusion can be made that at this agglomeration size, the drying time was sufficient to prevent the release of further volatile material. The presence of such bubbles around agglomerated metal salt material not only allowed conclusions to be drawn about volatile residues in the crystalline material, but also the presumption of the formation of a small oxidative atmosphere between the metal salt layer substrate and the Parylene layer. This trapped gas could, for example, consist of the dissolved water of crystallisation, not yet completely evacuated solvent residues (methanol), and air components such as nitrogen and oxygen. Some of these substances have the potential to reoxidise freshly reduced surface portions of the substrate material. These circumstances could lead, in some cases, to the result that the concretely targeted oxidation state of the metal salt material is at least partially changed via reoxidation processes by the formation of mixed phases of the most diverse oxidation states. This can also result in changes in the properties of the metal salt interlayer and thus of the entire multilayer system.

## 4. Conclusions

In this work, a new way of depositing metallic thin films in combination with Parylene-based barrier layers as a Parylene–metal multilayer system from solution was examined. It was found that the multilayer system resulted in different concentrations of two-layer components when applied in the same way. One was the uniformly covering base layer, which was approx. 1–2 nm thick and formed independently of the concentration. The other layer component consisted of larger crystal agglomerates, which occurred more frequently with increasing concentrations of the metal salt solution. Their size was measured between 10 and 30 nm. The surface roughness values were 4–8 nm for the metal salt layers and 20–30 nm for the surface of the Parylene layers, as an arithmetic mean. The orthogonal peel strength was between 4.4 and 4.7 kN. Despite the partially inhomogeneous formation of the metal salt layers, the application of a plasma-modified metal-containing thin film from a metal salt solution and subsequent Parylene coating provided a resource saving method to a one-pot synthesis of Parylene–metal multilayer systems. Future applications of these multilayer system production process could be in the time and cost-efficient creation of flexible wireless power and data transfer devices, as well as for UV-protective Parylene coatings, or mobile and durable catalytic surfaces, which can be applied in difficult geometric situations.

## Figures and Tables

**Table 1:** Sample types used for the multilayer coating systems.

	Type 1	Type 2	Type 3	Type 4
Glass slide	yes	yes	yes	yes
Metal salt layer	yes	yes	yes	yes
Varigon plasma	no	yes	no	yes
Parylene layer	no	no	yes	yes

**Table 2:** Determined Parylene C layer thicknesses by ellipsometric measurements, compared with the precursor weight.

Parylene C Precursor Weight (g)	Parylene C Layer Thickness (µm)
10.05	4.579
10.05	4.716
10.05	4.687
10.05	4.714

**Table 3:** Determined Parylene C layer thicknesses by white light reflectometry measurements compared with the precursor weight.

Parylene C Precursor Weight (g)	Parylene C Layer Thickness (µm)
9.914	5.825
9.987	5.739
9.997	6.304
10.09	5.742

## Data Availability

Not applicable.
